# Transcriptome-wide selection of a reliable set of reference genes for gene expression studies in potato cyst nematodes (*Globodera* spp.)

**DOI:** 10.1371/journal.pone.0193840

**Published:** 2018-03-02

**Authors:** Michael Sabeh, Marc-Olivier Duceppe, Marc St-Arnaud, Benjamin Mimee

**Affiliations:** 1 Saint-Jean-sur-Richelieu Research and Development Centre, Agriculture and Agri-Food Canada, St-Jean-sur-Richelieu, Quebec, Canada; 2 Biodiversity Centre, Institut de recherche en biologie végétale, Université de Montréal and Jardin botanique de Montréal, Montreal, Quebec, Canada; 3 Ottawa Laboratory Fallowfield, Canadian Food Inspection Agency, Ottawa, Ontario, Canada; University of Guelph, CANADA

## Abstract

Relative gene expression analyses by qRT-PCR (quantitative reverse transcription PCR) require an internal control to normalize the expression data of genes of interest and eliminate the unwanted variation introduced by sample preparation. A perfect reference gene should have a constant expression level under all the experimental conditions. However, the same few housekeeping genes selected from the literature or successfully used in previous unrelated experiments are often routinely used in new conditions without proper validation of their stability across treatments. The advent of RNA-Seq and the availability of public datasets for numerous organisms are opening the way to finding better reference genes for expression studies. *Globodera rostochiensis* is a plant-parasitic nematode that is particularly yield-limiting for potato. The aim of our study was to identify a reliable set of reference genes to study *G*. *rostochiensis* gene expression. Gene expression levels from an RNA-Seq database were used to identify putative reference genes and were validated with qRT-PCR analysis. Three genes, *GR*, *PMP-3*, and *aaRS*, were found to be very stable within the experimental conditions of this study and are proposed as reference genes for future work.

## Introduction

Quantitative reverse transcription PCR (qRT-PCR) is the most commonly used technique to measure the expression level of a particular gene and is considered to be the most accurate and reliable method so far [1,2]. However, sample preparation, from RNA extraction to complementary DNA (cDNA) synthesis, can introduce biases in the quantification. To overcome these sources of variation, reference genes are often used as internal controls to normalize the expression data [3]. However, it is now recognized that conventional housekeeping genes used as references (e.g., glyceraldehyde-3-phosphate dehydrogenase and β-actin) are not systematically appropriate, owing to their variability in some conditions [4–6]. It is therefore important to select and validate good reference genes based on their expression stability within all the experimental conditions, in order to ensure valid results [1].

Until now, microarrays were frequently used to test large numbers of genes simultaneously for the selection of good reference genes. Although this is not a bad approach, the technique requires previous knowledge of the nucleotide sequences of each candidate gene. With the advent of next-generation sequencing and, especially, RNA sequencing (RNA-Seq), this problem is now overcome, and quantifying a large number of gene transcripts without previous knowledge of their gene sequences can be done routinely. The RNA-Seq method yields millions of reads that can be assembled to generate a transcript database, which contains the sequences and expression levels of all expressed genes at a given time. Because RNA-Seq is quantitative, it can be used to study RNA expression [7,8]. Therefore, analyzing RNA-Seq data from different treatments should allow the identification of reliable reference genes for further qRT-PCR analyses, a strategy that has rarely been used to date, and to our knowledge, never applied to *Globodera* spp.

The potato cyst nematodes (PCNs), *Globodera rostochiensis* Wollenweber and *G*. *pallida* Stone, are plant-parasitic nematodes that affect exclusively Solanaceae plants, including potato (*Solanum tuberosum* L.), tomato (*S*. *lycopersicum* L.), and eggplant (*S*. *melongena* L.) [9]. Present in over 75 countries around the world [10] and recognized as quarantine organisms, PCNs limit plant growth and are among the most economically damaging nematodes [11]. They are responsible for estimated yield losses of 9% of the world’s production of potatoes, currently the fifth most important crop in the world, with an annual production of more than 368 million metric tons (FAOSTAT, 2015 [12]).

The nematodes enter the roots of the host plant, where they transform one of the plant cells to create a complex feeding site to pump plant nutrients. This will limits plant growth and eventually causes heavy yield loss. After maturation and fecundation, PCN females will dry to form a cyst containing up to 500 eggs. Inside their protective shell, these eggs can survive more than 20 years in the soil without a suitable host [13,14]. Because of the strength and durability of the cysts, PCN populations are difficult to control with currently available control strategies [15]. Hatching, host penetration, and establishment of the feeding cell are key stages of the life cycle that we need to focus on in order to find new PCN control methods. Studying gene expression during these stages could highlight essential genes against which control strategies could be developed. Therefore, we need a good technique to estimate gene expression levels during these key stages, as well as a reliable set of reference genes with constant expression across the experiment.

Many studies have been published reporting reference genes to analyze the expression of the plant gene transcripts, such as in potato roots infected with *G*. *pallida* [16] and in giant cells and syncytia induced by *Meloidogyne incognita* and *Heterodera schachtii* [17], but to our knowledge, no studies have yet focused on finding reference genes for PCN species. The aims of our study were to identify a set of potential reference genes for *G*. *rostochiensis* based on expression data obtained by RNA-Seq and to test reference genes previously reported in *Caenorhabditis elegans* for their expression in *G*. *rostochiensis*. In addition, the gene expression stability of the selected candidates was also evaluated in *G*. *pallida*.

## Materials and methods

### RNA-Seq dataset

This study took advantage of a recently published transcriptome dataset from a *G*. *rostochiensis* hatching experiment conducted by our team [18]. These data (NCBI bioproject accession number PRJNA274143) include sampling at different life stages, including dry cyst, hydrated cyst, hydrated cyst soaked in PRD for 1 h, 8 h, 24 h, 48 h, and 7 d, and fully hatched infective larvae (J2). Library preparation and sequencing were performed at McGill University and Génome Québec Innovation Centre (Montreal, Quebec, Canada) using the TruSeq RNA sample prep kit v2 (Illumina) and a HiSeq 2000 sequencer (Illumina, San Diego, California, United States). All eight samples were multiplexed and sequenced in one lane for 100 bp paired-end reads. Two replicates were processed and assembled into a *de novo* transcriptome using the Trinity assembler (for details, see Duceppe et al. [18]).

### Selection of candidate reference genes

A set of 15 genes previously used as references in the model nematode *C*. *elegans* [3,19,20] were selected to be evaluated in *G*. *rostochiensis*. These genes were *pmp-3*, *Y45F10D*.*4*, *tba-1*, *cdc-42*, *csq-1*, *eif-3*, *ama-1*, *mdh-1*, *gpd-2*, *act-1*, *act-2*, *F35G12*.*2*, *rbd-1*, *rgs-6*, and *unc-16*. The nucleotide sequences of these genes were retrieved from GenBank [21] and searched in the *G*. *rostochiensis* RNA-Seq transcriptome using BLAST to identify orthologues. Only the genes expressed in all the experimental conditions were kept for further analyses. The RNA-Seq dataset was also screened to identify transcripts with constant expression values in all the experimental conditions. This measurement of stability was based on standard deviations and expression variation through treatments, using log-transformed quantiles from normalized data. Only the four most stable genes were kept for further analysis.

### Expression stability analysis of candidate reference genes

#### RNA extraction and cDNA synthesis

The nematode populations used in this study were from a greenhouse rearing of *G*. *rostochiensis* pathotype Ro1, initially isolated from St-Amable, Quebec, Canada (obtained from Guy Bélair, AAFC), and *G*. *pallida* pathotype Pa2/3 from Noirmoutier, Vendée, France (obtained from Éric Grenier, INRA). The experimental conditions used were the same as in Duceppe et al. [18]: dry cyst, hydrated cyst, hydrated cyst soaked in potato root diffusate (PRD) for 1 h, 8 h, 24 h, 48 h, and 7 d, and fully hatched infective larvae (J2). Each *G*. *rostochiensis* sample contained approximately 1000 cysts, while the *G*. *pallida* samples contained only approximately 100 cysts because of low availability. Each sample was homogenized in 650 μL of RLT Plus buffer (Qiagen, Hilden, Germany) with one 6-mm zirconium grinding bead and 200 μL of 1-mm zirconium beads using the PowerLyzer 24 Homogenizer (Mo Bio, Carlsbad, California, United States) before RNA extraction. Total RNA was extracted using the RNeasy Mini Kit (Qiagen) according to the manufacturer’s instructions. All samples were treated with DNase (DNase I, New England Biolabs, Ipswich, Massachusetts, United States). A 2100 Bioanalyzer system (Agilent Technologies, Santa Clara, California, United States) was used to assess RNA concentration and purity. First-strand cDNA was synthesized with the SuperScript II reverse transcriptase (Invitrogen, Carlsbad, California, United States) according to the manufacturer’s instructions, from 0.5 μg of total RNA and using oligo (dT)_18_. Three replicates were made for each treatment.

#### Primer design and qRT-PCR

Primers were designed using PrimerQuest tool (Integrated DNA Technologies, Inc., Coralville, Iowa, United States) based on the sequences retrieved from the *G*. *rostochiensis* RNA-Seq dataset. Target fragments lengths were designed between 84 and 130 bp. Primer information for the candidate reference genes is listed in Table 1. Reactions were prepared using QuantiTect SYBR Green PCR kit (Qiagen) and amplified on a Mx3000P qPCR System (Agilent Technologies) in a final volume of 20 μL according to the manufacturer’s instructions. Melting curve analyses were done following the amplification cycles in order to examine the specificity of the reactions. Amplification efficiencies were calculated with dry cysts using the Real-time PCR Miner algorithm (ver. 4.0) [22].

**Table 1:** Description of genes and primers used in this study.

Gene symbol	Selection	Gene description	Primer sequence (5ʹ-3ʹ) forward/reverse	Amplicon length (bp)	Amplification efficiency (%)
*PMP-3*	*C*.* elegans*^**1**^	Putative membrane transporter	CTGGTTGCTGAGCAGGATAA/GATGAAGCCCGATTGGTAGAA	102	83
*Y45F10D*.*4*	*C*.* elegans*	Putative iron-sulfur cluster assembly enzyme	CCAAGCAGCACTGAGTGATTA/CATGATCCGCCGGGTTTATT	116	84
*CSQ-1*	*C*.* elegans*	Calcium-binding protein	GGTTGTGTTCTTCAACGATGTG/ACCCTCAGCCTTTGTTCTTT	102	85
*EIF-3*	*C*.* elegans*	Eukaryotic initiation factor 3	CCGCCAGTCCTATGTCATTTA/CTTCTTCGGGTCGGTGTATTAT	130	87
*AMA-1*	*C*.* elegans*	AMAnitin resistant family member	CTCCAAGCTCTCCACGTTATT/GGCGAAGTTGGACTGTATGT	118	84
*MDH-1*	*C*.* elegans*	Malate dehydrogenase	GCTGGACAAATTGGCTATTCAC/GAATGTCGAGGAGAACGAGAAC	94	86
*Act-1*	*C*.* elegans*	Actin-1	TGTAACCCACACTGTACCAATC/TTCATGAGGTAGTCGGTCAAATC	99	89
*aaRS*	RNA-Seq^**2**^	Aminoacyl tRNA synthetase	CGGATTTACGGACCTTGTCTAC/GGGAATCCGTCACGCTTAAT	84	86
*mce1*	RNA-Seq	mRNA capping enzyme	CCCGCATAAACTCCCATCTT/CTTCACACCGATTTGCCTTTC	118	85
*GR*	RNA-Seq	Glutathione reductase	TTGAGAGACCATGCCGATTAC/GAGTTGAGACGCCGAATGT	101	80
*ArgRS*	RNA-Seq	Arginyl-tRNA synthetase	GCCAACGCAAGAACCTTTAC/GCGACGTCGGGATGATATTT	109	83
*NEP-1*	Validation^3^	Neprilysin NEP-1	GCTGAAATGGTGGAGAAAGTG/TTTGACGCCCGAGTAGAAG	457	82
*cht-2*	Validation	Chitinase	ACAACTATTATGGCGGAAGGAG/GGTGTTGAGTGAATCAGAAGGA	94	83
*eng*	Validation	β-endoglucanase	CTCATACCCACACGTTCTCTAC/TAGCCTGATTTGGACTTGGG	106	85

^1^ Reference genes previously reported in *C*. *elegans* and having orthologues in *G*. *rostochiensis*.

^2^ Contigs from the analysis of the *G*. *rostochiensis* RNA-Seq database with stable expression levels across all experimental conditions.

^3^ Genes known to have different levels of expression in *G*. *rostochiensis* and used to validate the candidate reference genes.

#### Data analysis

RefFinder [23], a wrapper tool that integrates the statistical algorithm of BestKeeper [24], NormFinder [25], and geNorm [26], and the ΔCt method [27], was used to compare and rank the tested candidate reference genes. Based on the rankings from each program, it assigns an appropriate weight to an individual gene and calculates the geometric mean of their weights for the overall comprehensive ranking. Gene expressions from both the RNA-Seq database (read numbers) and qRT-PCR data (Cq values) across all treatments were compared. The variability of each gene across all treatments and replicates was also directly observed by plotting the distribution of the raw Cq values from the qRT-PCR experiment.

### Validation of reference genes

Relative expression analyses of three genes with published expression data (*NEP-1*, *cht-2*, and *eng*) were performed using the 2^−ΔΔCt^ method [28] in order to validate the selected reference genes. The genes *GR*, *PMP-3*, and *aaRS* were used as a reference set to normalize the expression of the targeted genes. Dry cysts were treatment used as the calibrator to calculate the fold changes for the other treatments. Gene expression was also normalized using the *Act-1* gene for comparison.

## Results

### Selection of candidate reference genes

Among the 15 putative reference genes previously reported in *C*. *elegans* and selected for this study, 11 were found to have orthologues in *G*. *rostochiensis* (*PMP-3*, *Y45F10D*.*4*, *tba-1*, *cdc-42*, *CSQ-1*, *EIF-3*, *AMA-1*, *MDH-1*, *gpd-2*, *Act-1*, and *Act-2*). However, four of them (*tba-1*, *cdc-42*, *gpd-2*, and *act-2*) were eliminated because they had no expression values for at least one experimental condition in the RNA-Seq experiment (data not shown). Four contigs from the analysis of the RNA-Seq database (*aaRS*, *GR*, *mce1*, and *ArgRS*) were also selected as candidate reference genes because their expression levels were stable across all experimental conditions. Specific primers were designed for these genes (Table 1).

### Expression stability analysis of candidate reference genes in *G*. *rostochiensis*

**Fig 1:**
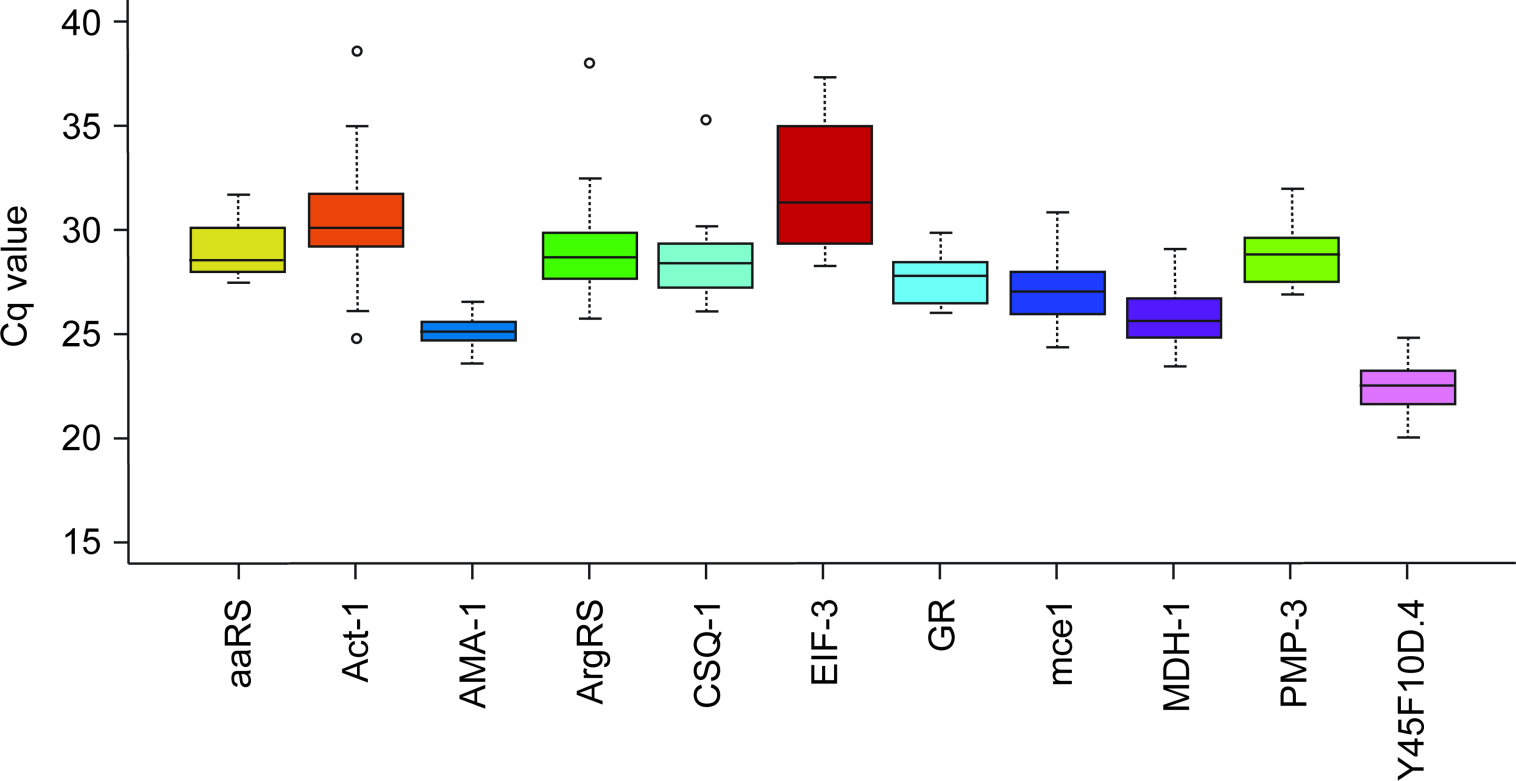
Variability of the expression level (span of Cq values) in *G. rostochiensis* of the 11 candidate reference genes across eight combinations of development stages and time of exposure to potato root diffusate, as measured by qRT-PCR. The median of three replicates of the eight conditions is represented by the line inside the box, 50% of the values are inside the box, the upper and lower edges represent the 75th and 25th percentiles, respectively, and the circles represent outliers.

**Fig 2:**
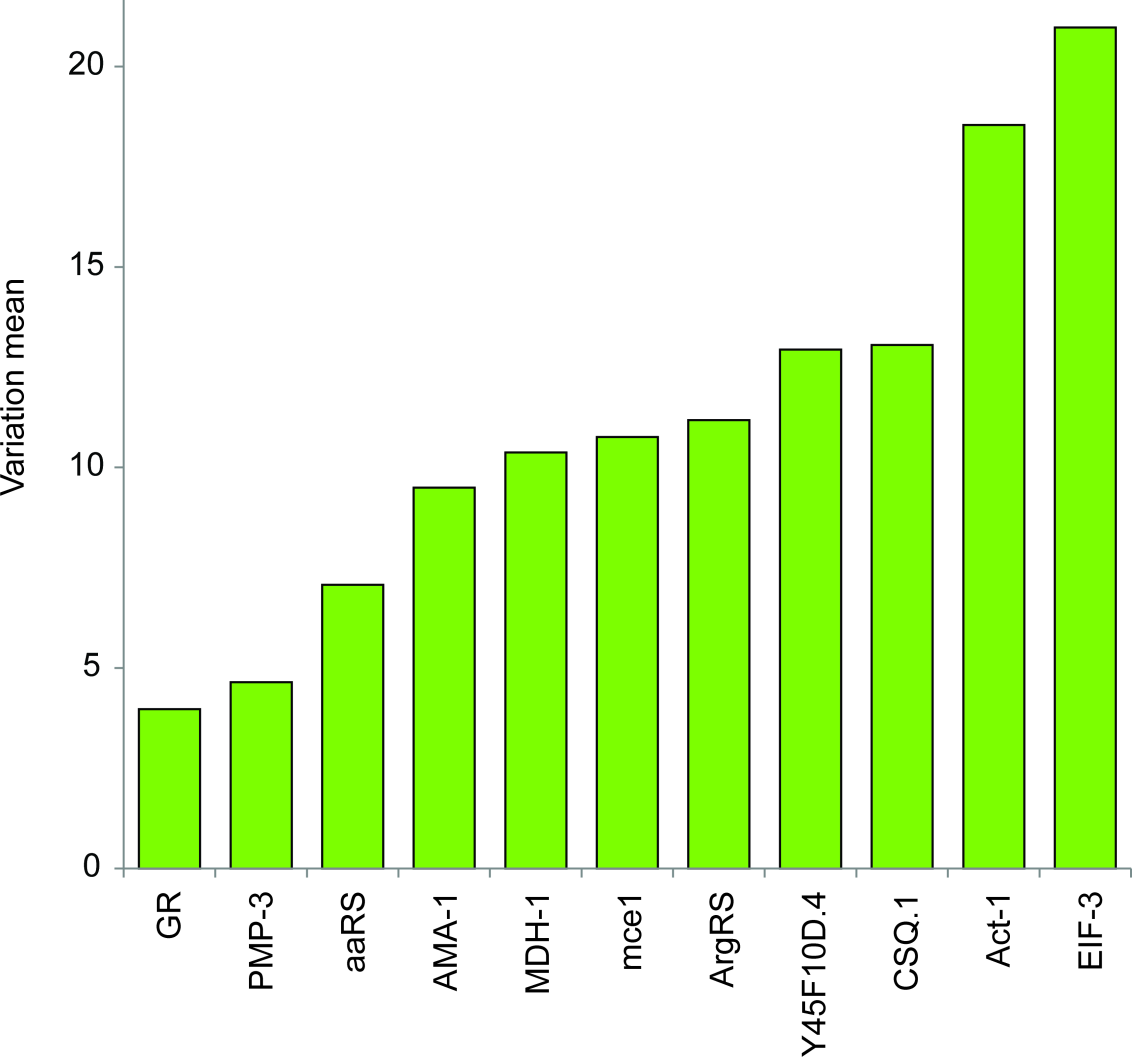
Summation of the comprehensive ranking values of gene stability given by RefFinder for RNA-Seq and RT-qPCR variation analyses for 11 candidate reference genes. Expression variations were calculated on two RNA-Seq replicates and three qRT-PCR replicates for the eight developmental stages of *G*. *rostochiensis*.

According to the distributions of raw Cq values, the genes with the lowest ranges were *GR*, *AMA-1*, *MDH-1*, and *aaRS*. The genes *EIF-3* and *Act-1* were the worst candidates, with Cq values spanning several units (Fig 1). However, a comparison of the distribution of the raw Cq values is not sufficient to evaluate the expression stability of candidate reference genes. Overall gene expression stability for both methods was assessed with RefFinder (Fig 2). *GR*, *PMP-3*, and *aaRS* expression were found to be the most stable, while *EIF-3*, *Act-1*, and *CSQ-1* showed the highest variability across treatments. Details of this ranking based on individual algorithms are given in Table 2. All four methods gave a roughly similar ranking except BestKeeper with the qRT-PCR data. The genes *GR, PMP-3*, and *aaRS* were rated among the four most stable genes across both methods.

**Table 2:** Comparison of rankings of 11 candidate reference genes from (A) RNA-Seq data and (B) qRT-PCR data, given by RefFinder in *G*. *rostochiensis*.

**A**	**ΔCt**	**BestKeeper**	**NormFinder**	**geNorm**	**Comprehensive ranking**	**Ranking**
	*GR*	*ArgRS*	*GR*	*ArgRS | PMP-3*	*GR*	**1**
	*aaRS*	*PMP-3*	*mce1*		*ArgRS*	**2**
	*PMP-3*	*aaRS*	*AMA-1*	*aaRS*	*PMP-3*	**3**
	*ArgRS*	*GR*	*aaRS*	*GR*	*aaRS*	**4**
	*mce1*	*MDH-1*	*PMP-3*	*MDH-1*	*mce1*	**5**
	*MDH-1*	*mce1*	*ArgRS*	*mce1*	*AMA-1*	**6**
	*AMA-1*	*AMA-1*	*MDH-1*	*AMA-1*	*MDH-1*	**7**
	*CSQ-1*	*Y45F10D*.*4*	*CSQ-1*	*Y45F10D*.*4*	*CSQ-1*	**8**
	*Y45F10D*.*4*	*CSQ-1*	*Y45F10D*.*4*	*CSQ-1*	*Y45F10D*.*4*	**9**
	*Act-1*	*Act-1*	*Act-1*	*Act-1*	*Act-1*	**10**
	*EIF-3*	*EIF-3*	*EIF-3*	*EIF-3*	*EIF-3*	**11**
**B**	**ΔCt**	**BestKeeper**	**NormFinder**	**geNorm**	**Comprehensive ranking**	**Ranking**
	*GR*	*Y45F10D*.*4*	*GR*	*aaRS | PMP-3*	*GR*	**1**
	*PMP-3*	*MDH-1*	*PMP-3*		*PMP-3*	**2**
	*AMA-1*	*GR*	*AMA-1*	*CSQ-1*	*AMA-1*	**3**
	*CSQ-1*	*Act-1*	*CSQ-1*	*AMA-1*	*aaRS*	**4**
	*aaRS*	*mce1*	*MDH-1*	*GR*	*Y45F10D*.*4*	**5**
	*MDH-1*	*AMA-1*	*aaRS*	*mce1*	*CSQ-1*	**6**
	*mce1*	*PMP-3*	*Y45F10D*.*4*	*Y45F10D*.*4*	*MDH-1*	**7**
	*Y45F10D*.*4*	*ArgRS*	*mce1*	*MDH-1*	*mce1*	**8**
	*ArgRS*	*CSQ-1*	*EIF-3*	*ArgRS*	*Act-1*	**9**
	*EIF-3*	*aaRS*	*ArgRS*	*EIF-3*	*ArgRS*	**10**
	*Act-1*	*EIF-3*	*Act-1*	*Act-1*	*EIF-3*	**11**

### Evaluation of reference genes in *G*. *pallida*

Candidate genes were also investigated in *G*. *pallida*, a closely related species. Both PCN species share the same host plants and have very similar morphological characteristics. The qRT-PCR analysis was performed using the same experimental design and primers that were designed for *G*. *rostochiensis*. The results for *G*. *pallida* were similar to those obtained for *G*. *rostochiensis*. Details of this ranking based on individual algorithms are given in Table 3. Based on the qRT-PCR analysis, *AMA-1*, *GR*, and *PMP-3* were the most stable potential reference genes for expression analysis in *G*. *pallida*. The results are similar to those obtained for *G*. *rostochiensis* as we find nearly the same genes in the first, second and last tier of the ranking.

**Table 3:** Detailed rankings of 11 candidate reference genes from qRT-PCR data, given by RefFinder in *G*. *pallida*.

ΔCt	BestKeeper	NormFinder	geNorm	Comprehensive ranking	Ranking
*AMA-1*	*PMP-3*	*GR*	*AMA-1*	*AMA-1*	**1**
*GR*	*aaRS*	*AMA-1*	*mce1*	*GR*	**2**
*mce1*	*GR*	*aaRS*	*GR*	*PMP-3*	**3**
*aaRS*	*Y45F10D*.*4*	*PMP-3*	*Y45F10D*.*4*	*mce1*	**4**
*Y45F10D*.*4*	*EIF-3*	*mce1*	*MDH-1*	*aaRS*	**5**
*PMP-3*	*AMA-1*	*Y45F10D*.*4*	*aaRS*	*Y45F10D*.*4*	**6**
*MDH-1*	*mce1*	*MDH-1*	*PMP-3*	*MDH-1*	**7**
*EIF-3*	*CSQ-1*	*EIF-3*	*EIF-3*	*EIF-3*	**8**
*CSQ-1*	*MDH-1*	*CSQ-1*	*CSQ-1*	*CSQ-1*	**9**
*Act-1*	*Act-1*	*Act-1*	*Act-1*	*Act-1*	**10**

### Validation of reference genes

**Fig 3:**
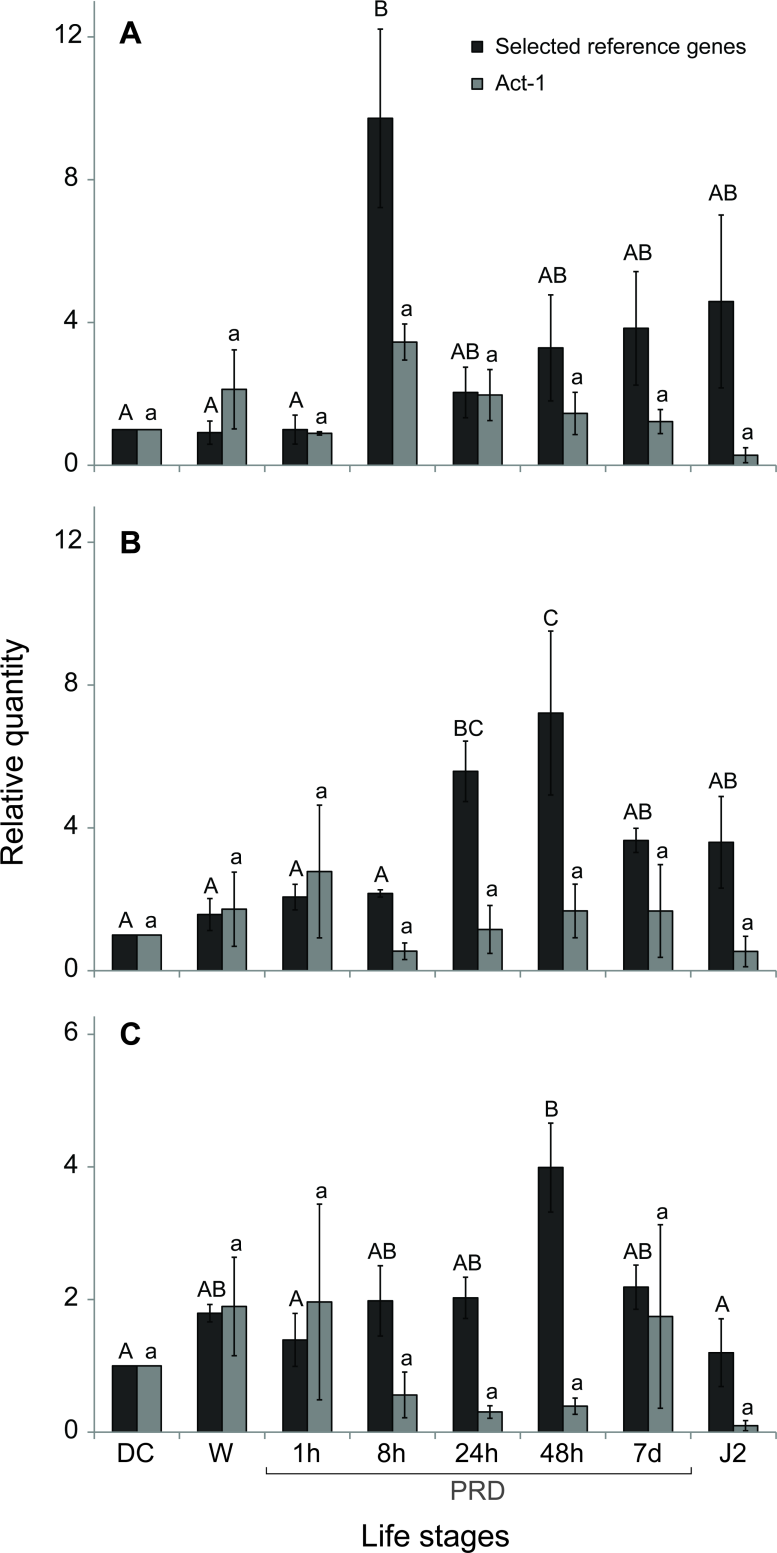
Expression of (A) *NEP-1*, (B) *cht-2*, and (C) *eng* assessed by qRT-PCR in dry cysts (DC), water hydrated eggs (W), hydrated eggs exposed to potato root diffusate (PRD) for 1 h, 8 h, 24 h, 48 h, and 7 d, and in J2 larvae. All expression levels were normalized using the geometric mean of *aaRS*, *PMP-3*, and *GR*, *our selected reference genes (in black) and Act-1 (in grey)*. Dry cyst was used as the calibrator for relative expression calculation. Error bars represent the standard error of the mean, and significant differences among treatments are indicated by different letters (Tukey’s test).

Validation of these recommended reference genes was performed using three genes (*NEP-1*, *cht-2*, and *eng*) with known patterns of expression. A qRT-PCR analysis was performed across the life stages of *G*. *rostochiensis* for each of these genes using the selected reference genes (*aaRS*, *PMP-3*, and *GR*) to normalize the data. The gene *NEP-1* was found to be overexpressed nearly 10 times after 8 h of exposure to PRD, *cht-2* was overexpressed 6 to 7 times after 24 to 48 h of exposure to PRD, and *eng* was overexpressed nearly 4 times after 48 h of exposure to PRD (Fig 3). In comparison, when normalization was performed using the *Act-1* gene, no significant difference was found in gene expression.

## Discussion

Even though the PCNs, *G*. *rostochiensis* and *G*. *pallida*, are major threats to agriculture worldwide, many aspects of the infection process remain largely unknown, and several plant-nematode genomics analyses are currently underway. However, a set of reliable reference genes for the normalization of gene expression in qRT-PCR studies is still lacking. A perfect reference genes should have a constant expression and be unaffected by the experimental treatments. Because this is rarely met in practice, the use of at least three reference genes is generally recommended in order to obtain reliable expression data [5,29]. Previous studies often used common housekeeping gene as reference genes. However, it is now recognized that some of them are not suitable owing to their variability [4–6]. In this study, we evaluated seven reference genes frequently used for *C*. *elegans* in addition to four other candidates selected from RNA-Seq expression data across a range of *G*. *rostochiensis* development stages and times of exposure to root exudates. This study demonstrates the feasibility of using RNA-seq expression data for the selection of RT-qPCR reference gene, as well as, to our knowledge, the first validation of reference gene published for PCNs. This work was carried out in parallel with an in-depth characterization of gene expression during hatching in *G*. *rostochiensis* which led to the identification of the NEP-1 gene in Duceppe et al. [18]. The lack of good reference genes adapted to cyst nematodes was hampering this kind of expression studies and although we already used these genes in the above-mentioned work [18], they need to be formally validated using recognized methods and on more genes.

Using RefFinder, the genes *GR* (glutathione reductase), *PMP-3* (putative ABC transporter), and *aaRS* (aminoacyl tRNA synthetase) were found to be the most stable among all the candidates. Therefore, they are the best available to be used as reference genes for normalization in qRT-PCR gene expression experiments for *G*. *rostochiensis* within the same experimental condition. Two of the three recommended reference genes (*GR* and *aaRS*) were identified based on RNA-Seq expression data analysis. This shows that RNA-Seq is an efficient approach to identify more stable and reliable reference genes and that public repositories like the NCBI Short Read Archive should be more exploited. The third gene, *PMP-3*, had already been selected as a reliable reference gene for *C*. *elegans* in previous studies [3,19]. Our results showed that this gene was also appropriate in *G*. *rostochiensis*, and may be a good candidate overall since its expression was found to be very stable in experimental conditions that differed noticeably from those generally found in most *C*. *elegans* studies. RNA-Seq data mining was found to be a very useful strategy to identify new reference genes from a much larger pool of candidates than qRT-PCR.

The *PMP-3* gene encodes a peroxisomal membrane protein putative ABC transporter [30], the *GR* gene encodes a glutathione reductase that is involved in oxidative stresses regulation [31], and the *aaRS* gene encodes an aminoacyl tRNA synthetase that is part of RNA translation [32]. Since none of these recommended reference genes belong to the same metabolic pathway, their combination decreases the probability that an experimental factor would affect the expression of all three reference genes, and therefore support their common use as a reference gene set.

The rankings given by the four calculation methods (ΔCt, BestKeeper, NormFinder, and geNorm) are slightly different because they use different algorithms and calculation approaches (Table 2). RefFinder proposes a comprehensive ranking that considers calculations from the four methods and was used here to select the reference genes. Some candidate reference genes that had been ranked at the top of the RNA-Seq analysis were not as stable when tested using qRT-PCR. This difference could be due to the sample preparation, in addition to the multiple bioinformatics steps required before the expression analysis could take place. Some genes that are routinely used as reference genes for other nematode species in the literature (e.g., *EIF-3* and *Act-1 [1,2,33]*) were less stable than those that were found as the best potential reference genes in the present study. This suggests that different morphological and physiological stages of *G*. *rostochiensis* may influence the expression of these usual reference genes. This influence would not be surprising, considering the huge reshuffling of gene expression across the different developmental stages, spanning from dormant cyst to infective juvenile larvae [18,34,35].

Very similar results were found with the sister species, *G*. *pallida*, for the ranking of reference gene candidates according to their stability. This similarity was expected, given the close phylogenetic relationship between *G*. *rostochiensis* and *G*. *pallida*. Using the same primers (for two out of three reference genes) for these two species would also increase the practicality of this set of reference genes.

To test the set of reference genes, we used it to normalize the expression of the *NEP-1*, *cht-2*, and *eng* genes in multiple life stages of *G*. *rostochiensis* known to have different levels of expression. The results were consistent with those previously reported. Duceppe *et al*. [18] showed that NEP-1 was significantly overexpressed 6.7 times after 8h of exposure to PRD; cht-2 transcripts were 4.9 and 5.3 times more abundant 24 and 48h after exposure to PRD respectively and eng showed a 4.4 fold change after 48h in PRD. These data are very similar to our RT-qPCR results after normalization with the proposed set of reference genes (Fig 3). In comparison, data normalization using the *Act-1* gene, revealed no significant difference in gene expression according to a Tukey’s test (Fig 3). This result is probably explained by the high variation in the *Act-1* Cq values across the treatments (Fig 1) confirming that this gene is not stable enough to be used as reference. This was also confirmed in other organisms, for example expression levels of actin were found to be highly affected by biotic or abiotic stresses in potato [36]. These observations further support the efficiency and purpose of our selected set of reference genes. The *NEP-1* gene, which is involved in the degradation of peptides and in post-transcriptional modification, was also found to be overexpressed prior to hatching in *C*. *elegans* [37]. The *cht-2* gene, codes for a hydrolytic enzyme that degrades chitin. In plant-parasitic nematodes, chitin has been found only in the eggshell and was found to be overexpressed during hatching [38]. The *eng* gene codes for beta-endoglucanase, a polysaccharide-degrading enzyme. That gene was also found to be expressed prior to hatching in *Globodera tabacum*, a closely related species [39]. In the present study, the normalized expression of these three genes was consistent with expectations from the previously reported studies. This confirms the efficiency of the selected reference genes.

## Conclusion

In this work, we showed that the expression of the genes *GR*, *PMP-3*, and *aaRS* was stable in all tested stages of the PCNs nematodes life cycle and duration of exposition of hydrated cysts in potato root exudates. We, therefore, recommend their use as reference genes in qRT-PCR analysis of PCNs nematodes. It is important however to remind that some life stages–J3 and J4 –were not tested in this work. Researchers willing to use these reference genes for sexual differentiation study, for example, should validate their stability in their experimental conditions. This study also demonstrated the benefit of using RNA-Seq expression data for the identification of novel candidate reference genes for qRT-PCR analyses as well as the concordance of the RNA-seq and RT-qPCR expression data.

## References

[1] Kozera B, Rapacz M. Reference genes in real-time PCR. J Appl Genet. 2013; 54: 391–406. doi: 10.1007/s13353-013-0173-x 24078518 10.1007/s13353-013-0173-x PMC3825189

[2] Radonić A, Thulke S, Mackay IM, Landt O, Siegert W, Nitsche A. Guideline to reference gene selection for quantitative real-time PCR. Biochem Biophys Res Commun. 2004; 313: 856–862. 14706621 10.1016/j.bbrc.2003.11.177

[3] Hoogewijs D, Houthoofd K, Matthijssens F, Vandesompele J, Vanfleteren JR. Selection and validation of a set of reliable reference genes for quantitative *sod* gene expression analysis in *C*. *elegans*. BMC Mol Biol. 2008; 9: 9 doi: 10.1186/1471-2199-9-9 18211699 10.1186/1471-2199-9-9 PMC2254638

[4] Dheda K, Huggett JF, Chang JS, Kim LU, Bustin SA, Johnson MA, et al The implications of using an inappropriate reference gene for real-time reverse transcription PCR data normalization. Anal Biochem. 2005; 344: 141–143. doi: 10.1016/j.ab.2005.05.022 16054107 10.1016/j.ab.2005.05.022

[5] Huggett J, Dheda K, Bustin S, Zumla A. Real-time RT-PCR normalisation; strategies and considerations. Genes Immun. 2005; 6: 279–284. doi: 10.1038/sj.gene.6364190 15815687 10.1038/sj.gene.6364190

[6] Bustin SA, Nolan T. Pitfalls of quantitative real-time reverse-transcription polymerase chain reaction. J Biomol Tech. 2004; 15: 155–166. 15331581 PMC2291693

[7] Nagalakshmi U, Wang Z, Waern K, Shou C, Raha D, Gerstein M, et al The transcriptional landscape of the yeast genome defined by RNA sequencing. Science. 2008; 320: 1344–1349. doi: 10.1126/science.1158441 18451266 10.1126/science.1158441 PMC2951732

[8] Wang Z, Gerstein M, Snyder M. RNA-Seq: A revolutionary tool for transcriptomics. Nat Rev Genet. 2009; 10: 57–63. doi: 10.1038/nrg2484 19015660 10.1038/nrg2484 PMC2949280

[9] Bélair G. Les nématodes, ces anguillules qui font suer les plantes… par la racine. Phytoprotection. 2005; 86: 65–69.

[10] Yu Q, Ye W, Sun F, Miller S. Characterization of *Globodera rostochiensis* (Tylenchida: Heteroderidae) associated with potato in Quebec, Canada. Can J Plant Pathol. 2010; 32: 264–271.

[11] Jones JT, Haegeman A, Danchin EGJ, Gaur HS, Helder J, Jones MGK, et al Top 10 plant-parasitic nematodes in molecular plant pathology. Mol Plant Pathol. 2013; 14: 946–961. doi: 10.1111/mpp.12057 23809086 10.1111/mpp.12057 PMC6638764

[12] Food and Agriculture Organization. FAO Statistical Pocketbook 2015: Food & Agriculture Orgn; 2016.

[13] Turner SJ. Population decline of potato cyst nematodes (*Globodera rostochiensis*, *G*. *pallida*) in field soils in Northern Ireland. Ann Appl Biol. 1996; 129: 315–322.

[14] Den Nijs L, Karssen G. *Globodera rostochiensis* and *Globodera pallida*. EPPO Bull. 2004; 34: 309–314.

[15] Chauvin L, Caromel B, Kerlan M-C, Rulliat E, Fournet S, Chauvin J-É, et al Control of potato cyst nematodes *Globodera rostochiensis* and *Globodera pallida*. Cahiers Agric. 2008; 17: 368–374.

[16] Castro-Quezada P, Aarrouf J, Claverie M, Favery B, Mugniéry D, Lefebvre V, et al Identification of reference genes for normalizing RNA expression in potato roots infected with cyst nematodes. Plant Mol Biol Rep. 2013; 31: 936–945.

[17] Hofmann J, Grundler FMW. Identification of reference genes for qRT-PCR studies of gene expression in giant cells and syncytia induced in *Arabidopsis thaliana* by *Meloidogyne incognita* and *Heterodera schachtii*. Nematology. 2007; 9: 317–323.

[18] Duceppe M-O, Lafond-Lapalme J, Palomares-Rius JE, Sabeh M, Blok V, Moffett P, et al Analysis of survival and hatching transcriptomes from potato cyst nematodes, *Globodera rostochiensis* and *G*. *pallida*. Sci Rep. 2017; 7: 3882 doi: 10.1038/s41598-017-03871-x 28634407 10.1038/s41598-017-03871-x PMC5478601

[19] Zhang Y, Chen D, Smith MA, Zhang B, Pan X. Selection of reliable reference genes in *Caenorhabditis elegans* for analysis of nanotoxicity. PLoS ONE. 2012; 7: e31849 doi: 10.1371/journal.pone.0031849 22438870 10.1371/journal.pone.0031849 PMC3305280

[20] Taki FA, Zhang B. Determination of reliable reference genes for multi-generational gene expression analysis on *C*. *elegans* exposed to abused drug nicotine. Psychopharmacology. 2013; 230: 77–88. doi: 10.1007/s00213-013-3139-0 23681163 10.1007/s00213-013-3139-0 PMC3795882

[21] Benson DA, Karsch-Mizrachi I, Lipman DJ, Ostell J, Sayers EW. GenBank. Nucleic Acids Res. 2009; 37: D26–D31. doi: 10.1093/nar/gkn723 18940867 10.1093/nar/gkn723 PMC2686462

[22] Zhao S, Fernald RD. Comprehensive algorithm for quantitative real-time polymerase chain reaction. J Comput Biol. 2005; 12: 1047–1064. doi: 10.1089/cmb.2005.12.1047 16241897 10.1089/cmb.2005.12.1047 PMC2716216

[23] Xie F, Xiao P, Chen D, Xu L, Zhang B. miRDeepFinder: A miRNA analysis tool for deep sequencing of plant small RNAs. Plant Mol Biol. 2012; 80: 75–84. 10.1007/s11103-012-9885-2 22290409

[24] Pfaffl MW, Tichopad A, Prgomet C, Neuvians TP. Determination of stable housekeeping genes, differentially regulated target genes and sample integrity: BestKeeper–Excel-based tool using pair-wise correlations. Biotechnol Lett. 2004; 26: 509–515. 15127793 10.1023/b:bile.0000019559.84305.47

[25] Andersen CL, Jensen JL, Ørntoft TF. Normalization of real-time quantitative reverse transcription-PCR data: A model-based variance estimation approach to identify genes suited for normalization, applied to bladder and colon cancer data sets. Cancer Res. 2004; 64: 5245–5250. doi: 10.1158/0008-5472.CAN-04-0496 15289330 10.1158/0008-5472.CAN-04-0496

[26] Vandesompele J, De Preter K, Pattyn F, Poppe B, Van Roy N, De Paepe A, et al Accurate normalization of real-time quantitative RT-PCR data by geometric averaging of multiple internal control genes. Genome Biol. 2002; 3: Research0034 12184808 10.1186/gb-2002-3-7-research0034 PMC126239

[27] Silver N, Best S, Jiang J, Thein SL. Selection of housekeeping genes for gene expression studies in human reticulocytes using real-time PCR. BMC Mol Biol. 2006; 7: 33 doi: 10.1186/1471-2199-7-33 17026756 10.1186/1471-2199-7-33 PMC1609175

[28] Livak KJ, Schmittgen TD. Analysis of relative gene expression data using real-time quantitative PCR and the 2−ΔΔCT method. Methods. 2001; 25: 402–408. doi: 10.1006/meth.2001.1262 11846609 10.1006/meth.2001.1262

[29] Derveaux S, Vandesompele J, Hellemans J. How to do successful gene expression analysis using real-time PCR. Methods. 2010; 50: 227–230. doi: 10.1016/j.ymeth.2009.11.001 19969088 10.1016/j.ymeth.2009.11.001

[30] Li S, Armstrong CM, Bertin N, Ge H, Milstein S, Boxem M, et al A map of the interactome network of the metazoan *C*. *elegans*. Science. 2004; 303: 540–543. doi: 10.1126/science.1091403 14704431 10.1126/science.1091403 PMC1698949

[31] Mannervik B. The enzymes of glutathione metabolism: An overview. Biochem Soc Trans. 1987; 15: 717–718. 3315772 10.1042/bst0150717

[32] The C. elegans Sequencing Consortium. Genome Sequence of the Nematode *C*. *elegans*: A Platform for Investigating Biology. 1998; 282: 2012–2018. 10.1126/science.282.5396.2012 9851916

[33] Espinola SM, Ferreira HB, Zaha A. Validation of suitable reference genes for expression normalization in *Echinococcus* spp. larval stages. PLoS ONE. 2014; 9: e102228 doi: 10.1371/journal.pone.0102228 25014071 10.1371/journal.pone.0102228 PMC4094502

[34] Eves-van den Akker S, Laetsch DR, Thorpe P, Lilley CJ, Danchin EG, Da Rocha M, et al The genome of the yellow potato cyst nematode, *Globodera rostochiensis*, reveals insights into the basis of parasitism and virulence. Genome Biol. 2016; 17: 124 doi: 10.1186/s13059-016-0985-1 27286965 10.1186/s13059-016-0985-1 PMC4901422

[35] Cotton JA, Lilley CJ, Jones LM, Kikuchi T, Reid AJ, Thorpe P, et al The genome and life-stage specific transcriptomes of *Globodera pallida* elucidate key aspects of plant parasitism by a cyst nematode. Genome Biol. 2014; 15: R43 doi: 10.1186/gb-2014-15-3-r43 24580726 10.1186/gb-2014-15-3-r43 PMC4054857

[36] Nicot N, Hausman JF, Hoffmann L, Evers D. Housekeeping gene selection for real-time RT-PCR normalization in potato during biotic and abiotic stress. *J Exp Bot*. 2005; 56: 2907–2914. doi: 10.1093/jxb/eri285 16188960 10.1093/jxb/eri285

[37] Spanier B, Stürzenbaum SR, Holden-Dye LM, Baumeister R. *Caenorhabditis elegans* neprilysin NEP-1: An effector of locomotion and pharyngeal pumping. J Mol Biol. 2005; 352: 429–437. doi: 10.1016/j.jmb.2005.06.063 16081104 10.1016/j.jmb.2005.06.063

[38] Schwekendiek A, Maier TR, Womack CR, Byrne DJ, de Boer JM, Davis EL, et al Initial characterization of endochitinase genes of the plant-parasitic nematode *Heterodera glycines* [abstract]. J Nematol. 1999; 31: 568.

[39] Goellner M, Smant G, De Boer JM, Baum TJ, Davis EL. Isolation of beta-1,4-endoglucanase genes from *Globodera tabacum* and their expression during parasitism. J Nematol. 2000; 32: 154–165. 19270961 PMC2620441

